# An optimisation study of a solar tower receiver: the influence of geometry and material, heat flux, and heat transfer fluid on thermal and mechanical performance

**DOI:** 10.1016/j.heliyon.2021.e07489

**Published:** 2021-07-08

**Authors:** Hashem Shatnawi, Chin Wai Lim, Firas Basim Ismail, Abdulrahman Aldossary

**Affiliations:** a Mechanical Engineering Department, College of Engineering, Universiti Tenaga Nasional, 43000, Kajang, Selangor, Malaysia; b Power Generation Unit, Institute of Power Engineering (IPE), Universiti Tenaga Nasional, 43000, Kajang, Selangor, Malaysia; c Royal Commission for Jubail, Education Sector in Jubail (JTI & JIC), Jubail Industrial City, 31961, Saudi Arabia

**Keywords:** Solar tower power, External receiver, Longitudinal internal fins, Nanofluid, Liquid sodium, Thermal stress

## Abstract

The solar receiver is considered the cornerstone of the solar tower power system. In particular, it receives high-temperature heat flux rays, and extracts the maximum heat energy to be transferred to the heat transfer fluid, while minimising any thermal and mechanical stresses. Reducing the solar receiver size helps to reduce the loss of spillage; consequently, the thermal stress increases. Using a solar receiver with inserted triangular longitudinal fins enhances the heat transfer as well as strengthens the receiver tube. This study aims to optimise the number of fins, heat flux aiming point, heat transfer fluid, nanoparticle effect with molten salt as the base fluid, and type of receiver material. Non-uniform heat flux with the cosine and Gaussian effects have been considered. When the number of fins (N) increases, the maximum temperature (T_max_) decreases and the heat transfer is enhanced. When N = 20, T_max_ = 656.4 K and when N = 1, T_max_ = 683.55, while the efficiency for N = 1 is greater by 3% compared to when N = 20. The cosine distribution of heat flux has a higher maximum temperature than the Gaussian distribution by 29% and is 102% higher in receiver efficiency. The thermal efficiency when the heat flux is aimed at the middle point of the receiver is higher by 10% compared with a lower or upper aiming point. Using Al_2_O_3_ nanoparticles with a concentration of 0.5 wt.% increases the thermal efficiency by 14% more than when using pure molten salt when Re = 38000. Using liquid sodium is not required to monitor the peak heat flux, and by adding triangular fins the displacement and thermal stress are 6.5 % lower compared to a smooth receiver.

## 1 Introduction

Over the past decade, academic and engineering attention has been devoted to the solar receiver system due to the dramatic increase in fossil fuel prices and the detrimental environmental impact of fossil fuel use. The external receiver is considered a crucial component of the central solar system. One of the significant challenges in the design of tubular receivers is to find the correct configuration of various parameters, including receiver formation and geometry; receiver size and material; heat transfer fluid; and control of the maximum heat flux.

The radiation generated by the heliostat does not hit the receiver completely since the environment consumes a measure of energy. The collection failure, or spillage, is expressed in reflected energy from the mirrors aiming the receiver, which does not land on the absorption region [1]; reducing the receiver size significantly impacts the spillage loss. However, the advantages of small receiver sizes are highly valued, due to their better performance with a smaller heliostat and higher peak flux limit, reduced receiver costs, and improved thermal efficiency by reducing thermal losses such as convection and radiation [2].

The geometry of the solar receiver plays a vital role in heat transfer enhancement. As the receiver gets smaller, it can be supported by adding internal fins to reduce the temperature gradient, increase the internal heat transfer area in the receiver and improve heat transfer performance. Many studies have investigated the addition of internal fins along the interior surface of the parabolic trough solar receiver. However, several researchers have studied the insertion of fins in the solar tower receiver. Messaoud et al. [3], utilised helical fins to achieve a better heat transfer from the receiver to the heat transfer fluid, and a receiver with four helical fins was considered the best design. Liu et al. [4] developed a numerical model which used four different inserts with non-uniform heat flux. Twisted tapes had the highest Nusselt number and the lowest difference in circumference temperature. Further details about the use of internal fins are available in [5].

Heliostats can be used to precisely monitor the amount of sunlight that reaches a receiver's surface, enabling the receiver to be further tuned to increase the amount of sunlight absorbed. The cosine performance loss also is one of the most significant causes of energy depletion, which is calculated by the angle cosine of the vector from the surface of the reflector to the sun and the normal vector at the surface. As a cosine effect, a vast solar tower experiences sharp and non-uniform performance variance throughout the day [6].

Non-uniform flux distributions create high thermal stress on the receiver pipes, and are crucial in the design and operation of receiver systems [7]. Lowering heat flux gradients and reducing the peaks on the receiver can be an effective technique to improve the efficiency of the receiver. Various techniques have been used to reduce the maximum heat flux on the solar tower receiver by selecting different aiming points from heliostats. Binotti et al. [8], investigated various strategies for minimising peak heat flux on a solar receiver by adjusting the target points of the heliostats, and the overall heat flux decreased by 40 %.

The working conditions of the receiver are extremely challenging, since the temperature gradient is high and likely to produce significant thermal stresses [9]. The thermal stress that crosses the elastic limit of the material is a typical defect of the receiver tubes [10]. The influence of the circumferential absorbed flux variations and tube temperature fluctuations was not examined and this could be highly significant. A reduction of the temperature difference between the average and the front of the outer tube results in a reduction of the thermal stresses [11].

Peng et al., investigated the tube thickness (0.25 mm–2.2 mm) and the receiver diameter (12.4 mm–32.4 mm) effects on thermal stress; the effect of tube thickness was more significant than that of tube diameter in increasing thermal stress [12]. Ghomrassi et al. [13], numerically examined the efficiency of the tube receiver covered with a metallic coating for various diameters. Increasing the thickness of the metallic tube affects the receiver's heat transfer efficiency, and as the inner tube diameter decreases, the working fluid outlet temperature rises. Hazmooune et al. [14], investigated numerically different solar receiver thicknesses between 2 to 3mm; as the tube thickness increases the temperature gradient also increases. Furthermore, the highest temperature was found using alloy 625, while the lowest temperature was identified with copper.

The choice of heat transfer fluids has significant effects on the heat exchange performance and reliability of solar thermal systems. Liu et al., studied three types of heat transfer fluid with uniform and non-uniform heat flux. The heat transfer performance of liquid sodium provides promising results, where the heat transfer coefficient is higher than molten salt [15]. For sodium, it is unnecessary to lower the peak heat flux as in molten salt receivers. Even at high heat flux densities, the absorber tubes are sufficiently cooled due to the high heat transfer coefficients of sodium. Fritsch [16] increased the mean heat flux density by 200% and peak heat flux by 280% compared with molten salt receiver limits. William R. studied a solar receiver made from stainless steel 316 with liquid sodium as a heat transfer fluid, the thermoelastic stress was 35% lower compared with the receiver working with molten salt [17].

A simple cost-effective way to boost the heat transfer fluid in the solar receiver is to dope it with nanoparticles, which have stability and the appropriate heat capacity [18] **.** Zhaopin et al., examined the effect of molten salt with nanofluid based on the heat transfer of the solar receiver's performance with non-uniform heat flux. Compared with pure salt, a nanofluid base performs better on heat transfer enhancement [19]. Geng et al., simulated nanofluid systems with different weights of nanoparticles in sodium, potassium, and lithium nitrate salts. They found that adding nanoparticles increased the specific heat capacity of the heat transfer fluid [20].

This study is a continuation of previous work [5], The goal was to design a new solar receiver capable of outperforming the traditional receiver by minimising the thermal losses using a tiny diameter tube with internal fins to reduce the external area exposed to solar rays and to strengthen the receiver tube. A new molten salt receiver design was numerically investigated, following the addition of square, rectangular, circular, and triangular longitudinal fins with various heights. The triangular fin with a height of 1mm delivered the best heat transfer performance and the highest efficiency of the solar receiver compared with square, circular and rectangular fins, which is also reduced the inner wall temperature near the spot of maximum heat flux by 6%.

This paper aims to generate additional data through numerical analyses to develop the solar central receiver, and by exploring the effect of adding longitudinal triangular fins along the receiver tube on heat transfer performance. Consideration is given to the number of fins, receiver thickness and diameter, receiver materials, addition of nanoparticles, heat transfer fluid type, and heat flux distribution. Ultimately, the objective is a highly optimised solar tower receiver in terms of its thermal and mechanical features.

## 2 Physical model

### 2.1 Nonuniform heat transfer model of the solar receiver

**Figure 1:**
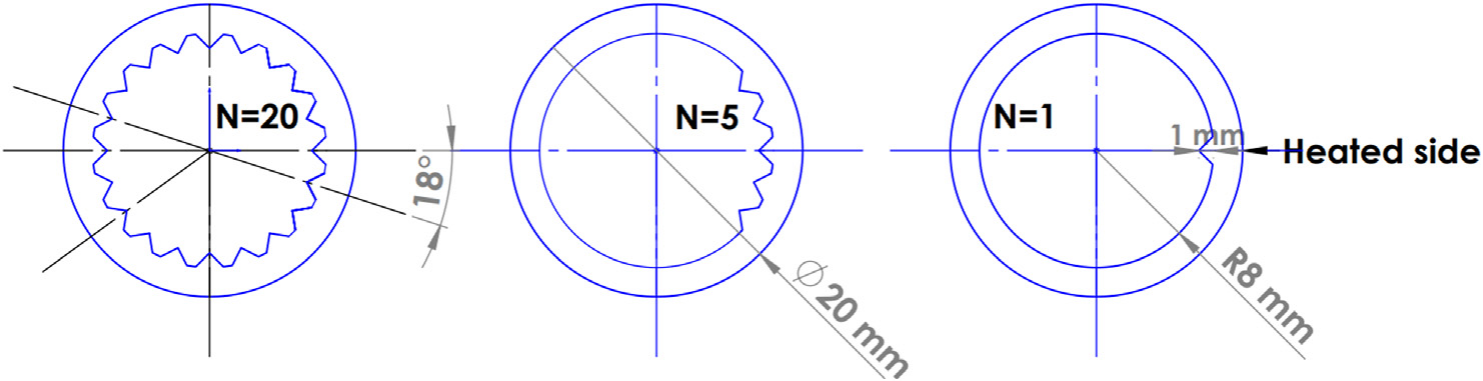
Receiver dimensions and fins distributions.

The cylindrical receiver comprises several panels in a cylindrical configuration, and each panel contained more than 20 thin-walled tubes. Tubes in the same panel have fluid flows in the same direction and have approximately the same flux distribution and similar boundary conditions. Since the collector is cylindrical we investigated only one single tube. The chosen dimension and working conditions were same as in [21]. The governing equations and boundary conditions are available in [5]**.** A tube made of Alloy 625 with a length of 1.3 m and a thickness of 2 mm was used. Half of the tube faced the heat flux and the other side was considered an adiabatic. Triangular fins with a height of 1mm were inserted longitudinally along the receiver. The number of fins (N) was between 1-20, as shown in Figure 1.

**Figure 2:**
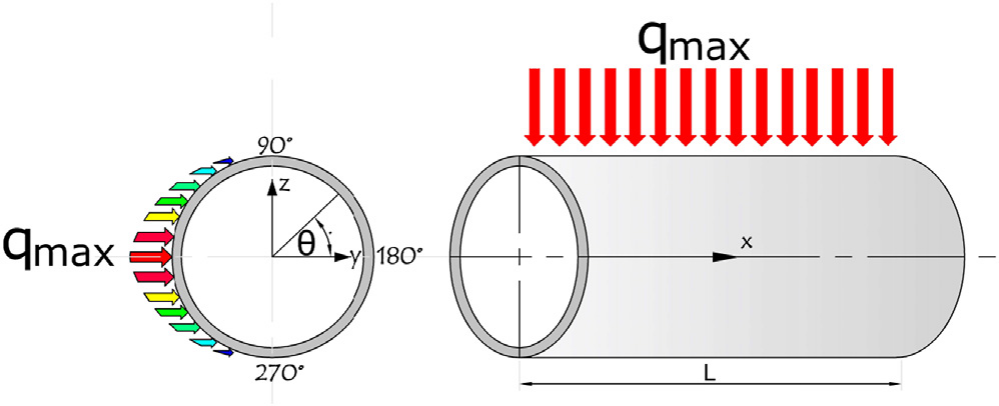
Heat flux distribution with neglecting the Gaussian effect.

**Figure 3:**
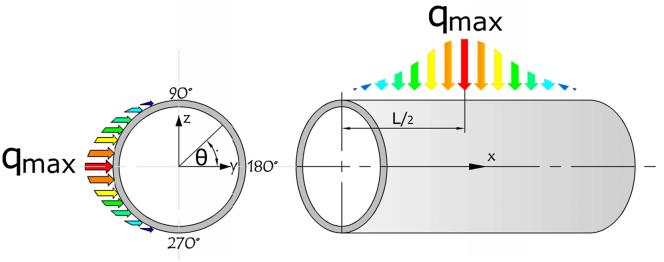
Heat flux distribution with considering the Gaussian effect.

Because the semi-circumference of the tube is heated with an uneven heat flux where the other semi-circumference is insulated, thus the heat flux follows either Cosine distribution, as shown in Figure 2, or the Gaussian law distribution [22] with its peak in the middle of the pipe's length, as shown in Figure 3.

Eq. (1) is used to express the heat flux distribution which neglected Gaussian distribution, while Eq. (2) considered the Gaussian effect, where *q*_*max*_ is the maximum heat flux [W/m^2^].

(1) $$\;q\left(x,\theta \right)=-q_{max}.\cos\left(\theta \right)\;;if\left(90\leq \theta \leq 270\right)$$

(2) $$q\left(x,\theta \right)=-q_{max}.\cos\left(\theta \right).f\left(x\right)\;;if\;\left(90\leq \theta \leq 270\right)$$

Gaussian law:

(3) $$f\left(x\right)=\text{exp}\left[-\frac{1}{2}{\left(\frac{xL-\mu }{\sigma }\right)}^{2}\right]$$

where:

$$\mu =\frac{L}{2},\;\sigma =\frac{L}{5},\;x=\frac{X}{L}:\;x=\left[0..L\right]$$

### 2.2 Non-dimensional quantities

Average Nusselt number is defined as

(4) $$Nu=\frac{D_{h}}{k}.\frac{\bar{q}}{\left({\bar{T}}_{w}-{\bar{T}}_{b}\right)}$$

(5) $$D_{h}=\frac{4A}{P}$$

where

*D*_*h*_: hydrulic diameter [m]; *A*: cross section area [m^2^]; P: perimeter [m]; *k*: molten Salt thermal conducvitiy [W/(m.K)]; $\bar{q}$: avergae Heat flux [W/m^2^]; ${\bar{T}}_{w}$: average wall temperature of inner wall; ${\bar{T}}_{b}$ : avergae bulk tempreture.

Reynolds number can be described as

(6) $$Re=\frac{\rho u_{in}D_{h}}{\mu }$$

where, *ρ*: molten salt density [Kg/m^3^], *μ*: salt viscosity [kg/m s], *u*_*in*_: inlet velocity [m/sec].

Dittus-Boelter correlation which is valid for Reynolds number greater than10,000 Prandtl number (*Pr*) between 0.7 to 120, and the ratio between length to diameter greater than 60

(7) $$Nu=0.023\;Re^{0.8}Pr^{0.3}$$

The receiver thermal performance parameter (η) of the modified external receiver is obtained from Eq. (8)

(8) $$\eta =\frac{Q}{q_{max}\;.\;A_{E}}=\frac{m_{f\;}c_{p}\left(T_{out}-T_{in}\right)}{q_{max}\;.\;A_{E}}$$

where Q is the rate of convection heat transfer in the test tube [W]. *A*_*E*_ is the external receiver area [m^2^] and *q*_*max*_ is the maximum applied heat flux [W/m^2^]. *m*_*f*_ is the mass flow rate of the molten salt in [kg/sec]. *C*_*p*_ is the specific heat capacity of molten salt in [J/kg K]. T_in_ and T_out_ are the inlet and outlet temperature respectively in [K].

### 2.3 Validation

**Figure 4:**
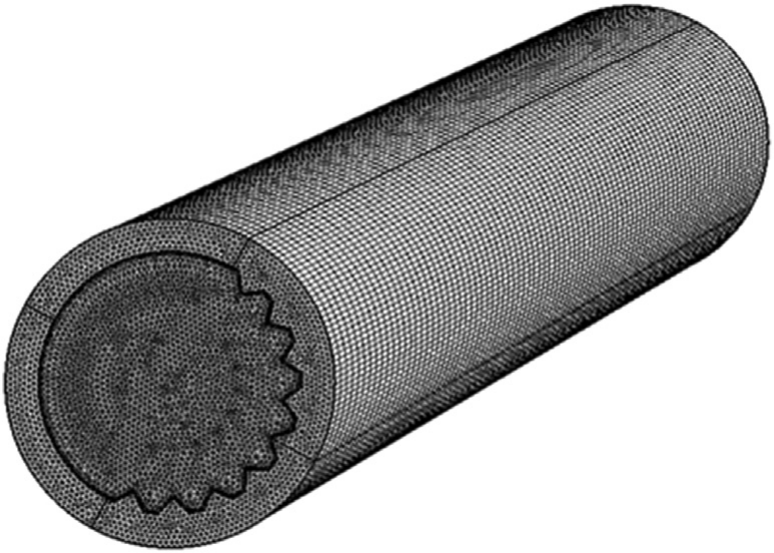
Finer mesh in 3D receiver tube.

To ensure the independence of the grid, nine mesh grids were tested, as provided by COMSOL, measuring from extremely coarse to extremely fine. With the number of elements equal to 920,000, the relative error in calculating the average outlet temperature was 1.5E-04 with the finer mesh, as illustrated in Figure 4. This was chosen as the optimal solution as any extra elements did not significantly alter the resulting temperatures.

**Figure 5:**
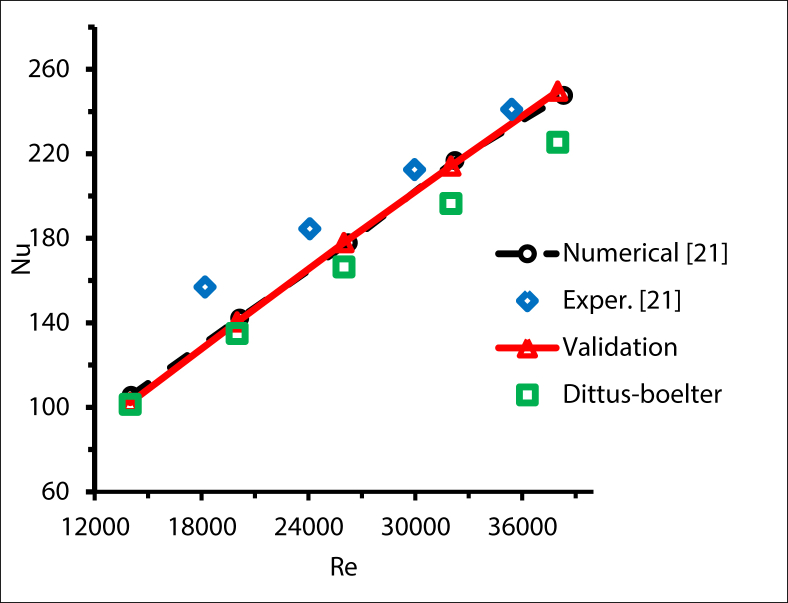
Validation of Nusselt numbers for a smooth tube, Re = 14000 to 38000.

Validation was conducted with a single tube under non-uniform heat flux. The obtained results compared with the experimental results, the numerical simulation and the Dittus-Boelter correlation and all the findings were consistent with the reference case. Composite nitrate salt is used as a thermal fluid in experiments. The molten salt was heated by a molten salt tank and furnace at a given temperature and transfers to an electric heater with a maximum power of 40 kW, and continued to be heated to the required experimental temperature. By modifying the output of the electric heater the constant heat flux on the recipient can be changed to the needed value. Figure 5 shows the validation test for a smooth tube with Reynolds between 14000 and 38000 and molten salt fluid.

## 3 Results and discussion

### 3.1 The effect of triangular fins

**Figure 6:**
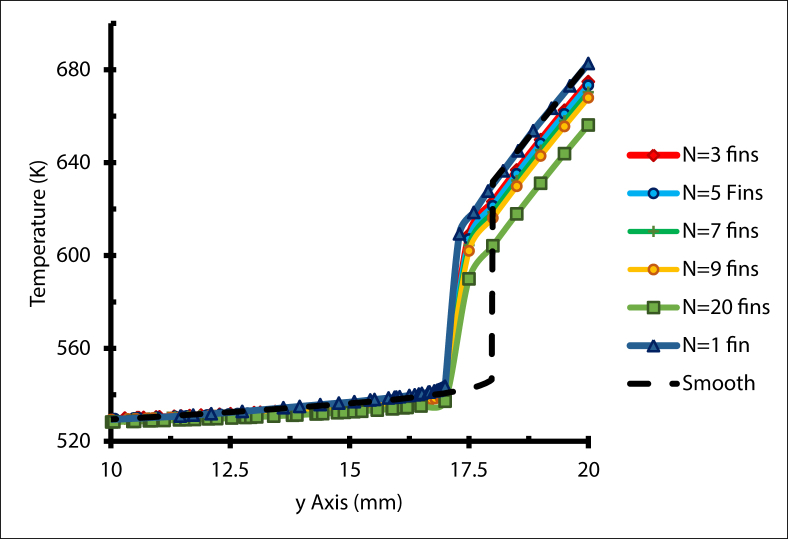
Temperature gradient at the receiver outlet, with Re = 14000.

Finned tubes are widely used for enhancing heat transmission [23]. Adding fins plays an important role in reducing the temperature gradient between the outer and the inner surface of the receiver, as the stress region is created by temperature gradients [24]. There was a sharp temperature gradient for the smooth receiver, as Figure 6 illustrates, while the slope of the temperature gradient decreased when the number of fins increased. In other words, inserting more fins has a significant influence on reducing the temperature gradient. Furthermore, adding longitudinal fins in a different location along the internal receiver circumference produced different flow shapes, and disturbed the boundary layer development. As a result, the flow rate and the turbulence rate rose. In addition, the hydraulic diameter decreased with the addition of more fins. Together, these worked to improve the convection rate and the overall heat transfer process.

**Figure 7:**
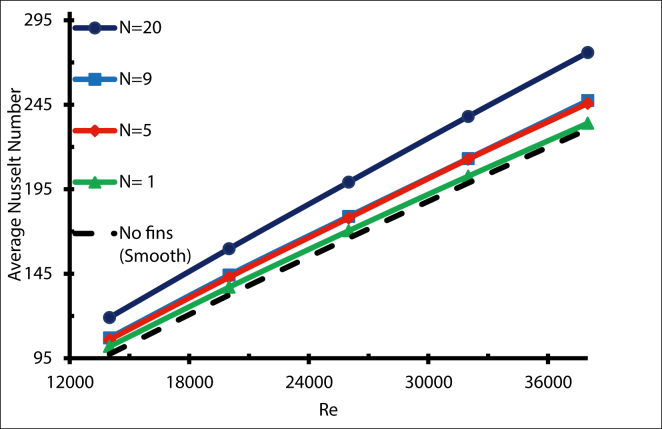
Average Nusselt number with different fins number.

Adding more fins enhanced the heat transfer process, which means the maximum value of the Nusselt number can be determined by adding more fins. Figure 7 illustrates the average Nusselt number of a receiver tube for different numbers of triangular fins, with a Reynolds number ranging from 14000 to 38000. For all values of N, as the Reynolds number increases, the Nusselt number rose as a result of higher convection. Compared with the smooth tube, the Nusselt number increment ratio when N = 1 is 4% when Re = 14000, and 1.5% when Re = 38000. Meanwhile, for N = 20 the increment ratio is 22% and 19 % for Re = 14000 and 38000, respectively.

A correlation was developed to predict the Nusselt number based on the number of fins (N) and Reynolds (Re), through the solar receiver with longitudinal triangular fins.

(9) $$Nu=\left(22.01979+\frac{-22.0142774}{1+{\left(\frac{N}{74598.15}\right)}^{1.218877}}\right)Re+\left(28.572+\frac{-2.612}{1+{\left(\frac{N}{9.208961}\right)}^{47.8647}}\right)$$

**Figure 8:**
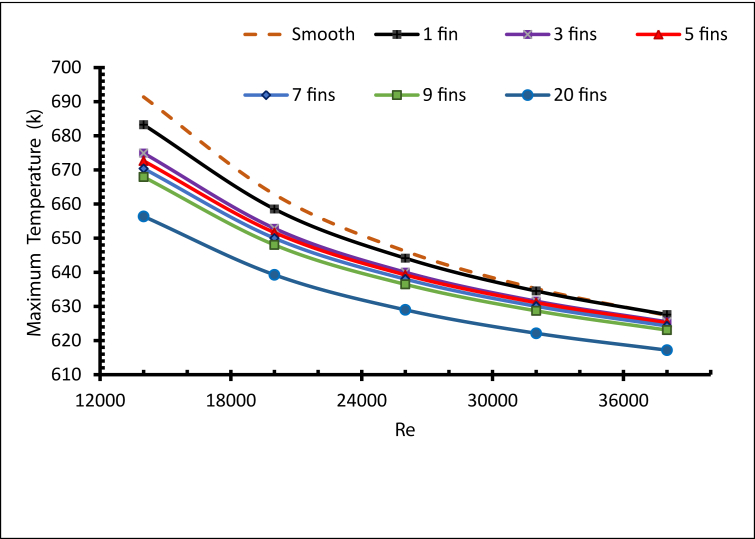
Maximum temperature with different fins number.

**Figure 9:**
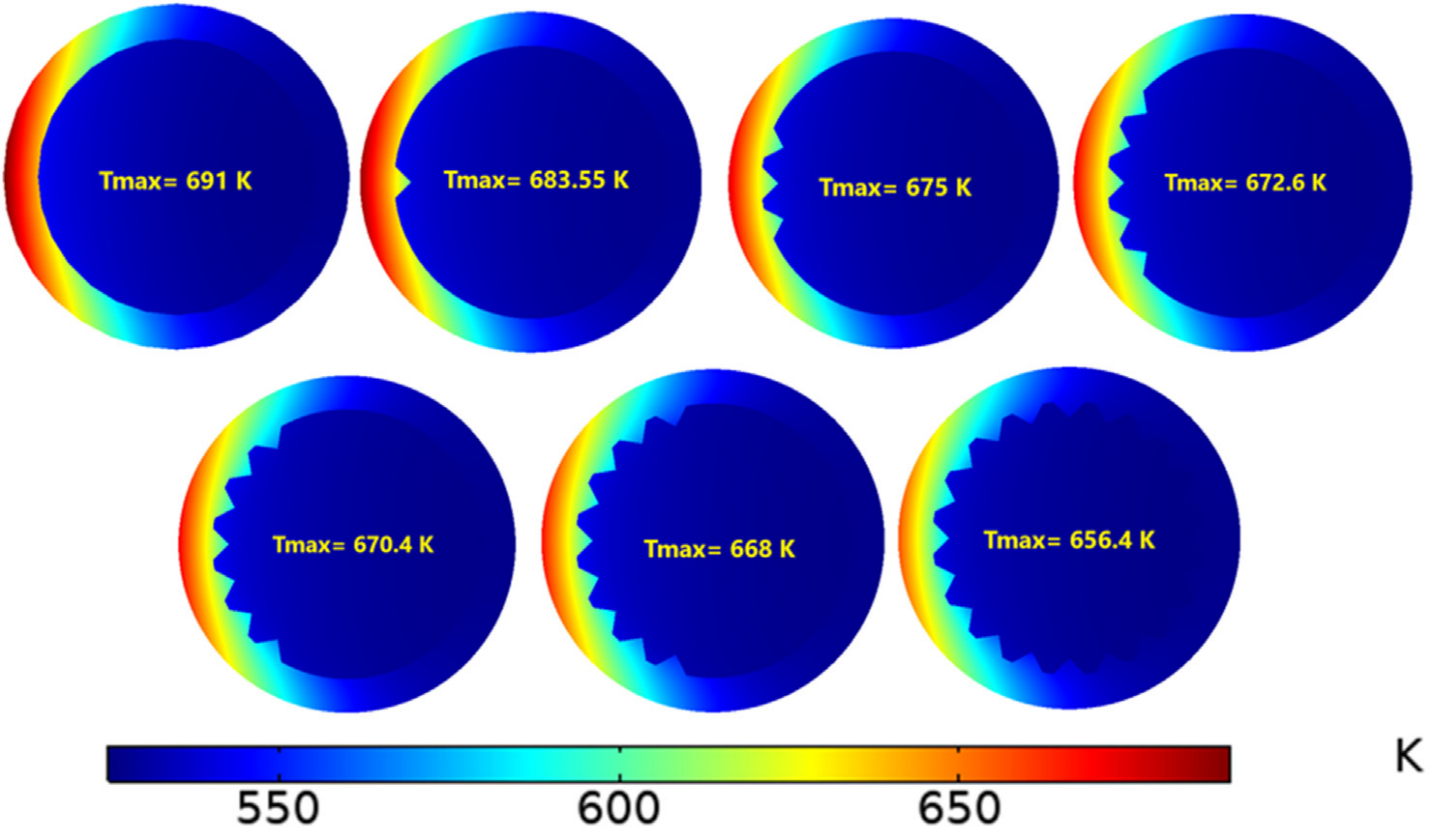
Peak Contour temperature.

Figure 8 demonstrates the maximum temperature of the receiver tube with different numbers of fins. Obviously, the maximum value of the temperature decreases as the Reynolds number rises, since the cooling of the outer surfaces improves with increased convection. On the other hand, fins significantly impacted the heat transfer and temperature development; they can enhance heat transfer from solid to fluid surfaces [25]. Adding further fins was reported to cause a drop in the peak temperature of the receiver tube [26]. The highest maximum temperature found for the smooth tube with Re = 14000 equalled 691 K. As the number of fins increased the maximum temperature decreased, and the lowest maximum temperature was 617 K when N = 20 and Re = 38000. As a result, cooling the receiver's outer surface with molten salt can be improved with additional fins, as presented by the temperature contours of the peak temperature in Figure 9, with N = 1,3,5,7,9 and 20.

**Figure 10:**
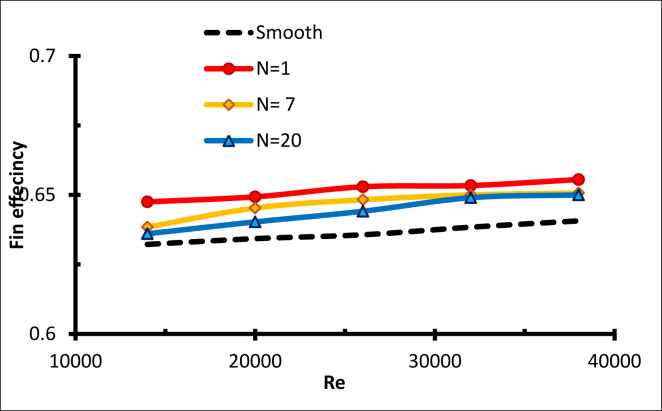
Receiver efficiency with different numbers of fins, N = 1,7 &20.

Another factor that should be considered when evaluating the receiver's performance after adding fins is the outlet temperature of the molten salt. The outlet temperature with fins is higher than the smooth tube [25], and this can be reflected in receiver efficiency (see Eq. 8), as shown in Figure 10. The efficiency is always higher with any number of fins compared with the smooth tube; these differences in efficiency illustrate the substantial impact of fins. The efficiency increases with a higher Reynolds number, and this is related to lower thermal losses and higher mass transfers with a high mass flow rate [26].

The maximum efficiency when N = 1 is around 66%. For high efficiency in the solar receiver, an equilibrium is required between the decrease in the hydraulic diameter, which enhances the convection rate, and the increase in residence time, which raises the outlet temperature. The optimal efficiency was found with N = 1.

### 3.2 The effect of heat flux

**Figure 11:**
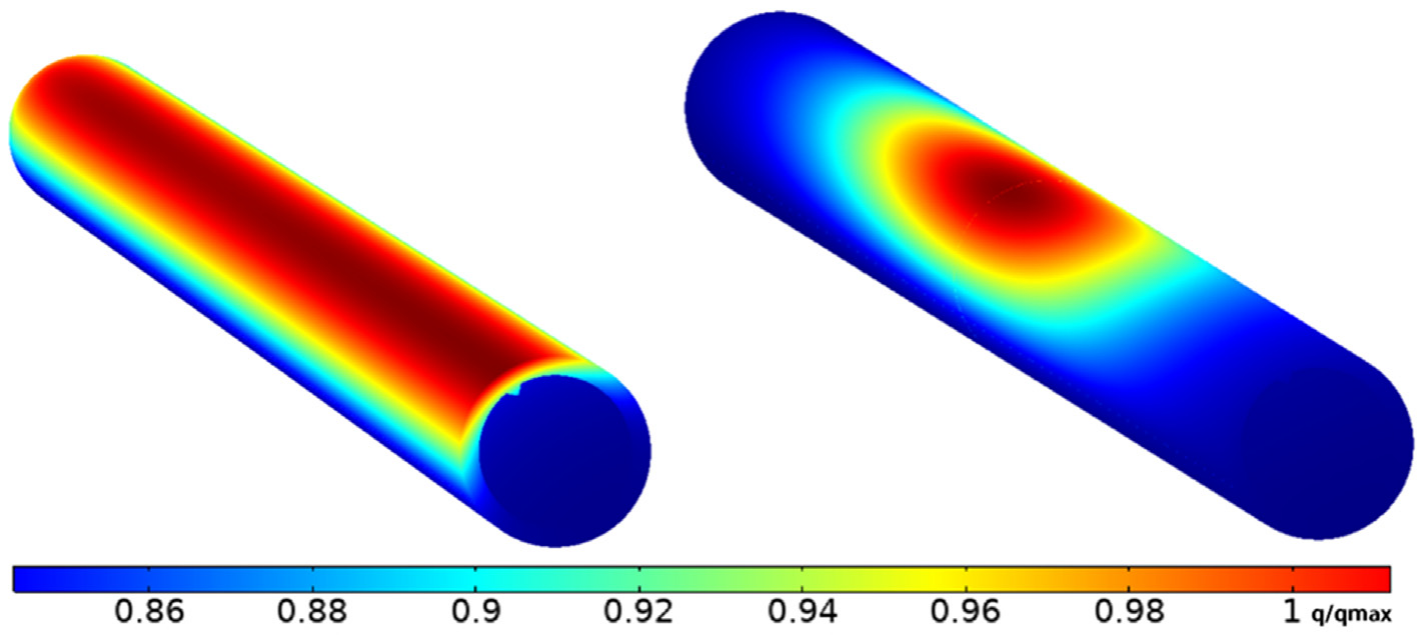
3D-Non-uniform heat flux distribution of the receiver tube.

**Figure 12:**
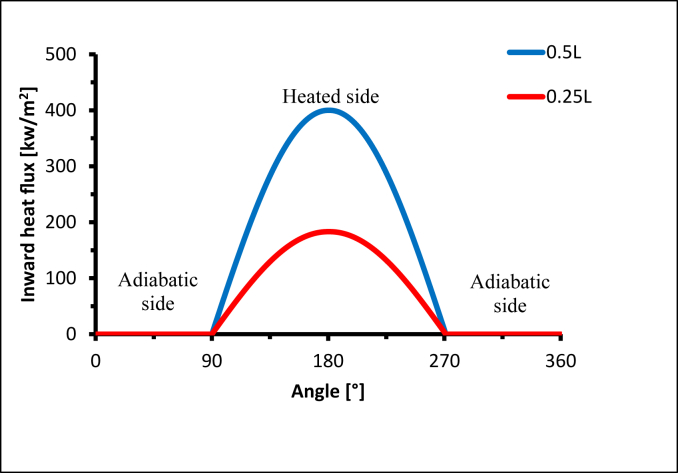
Axial inward heat flux along the receiver tube with Gaussian effect.

The concentrated solar flux distribution of the solar receiver is highly non-uniform. This can produce a wide temperature gradient and high local temperatures in solar receivers, which generate significant safety challenges and impair the efficiency of the receiver operation. To identify the optimum non-uniform heat flux distribution for both Cosine and Gaussian distribution with the consideration of inserting triangular fins, it was assumed that the aiming point was located at the centre of the receiver [8], as shown in Figure 11, and the heat flux was concentrated on the middle point of the receiver. For the Cosine distribution, the heat flux value remained constant along the axial line on the receiver's heated side; however, with the Gaussian effect, the value of heat flux changed along the axial line. It also decreased and moved away from the centre point of the receiver. The inward heat flux in x = 0.25L or x = 0.75L was lower by 118 %, as illustrated in Figure 12.

**Figure 13:**
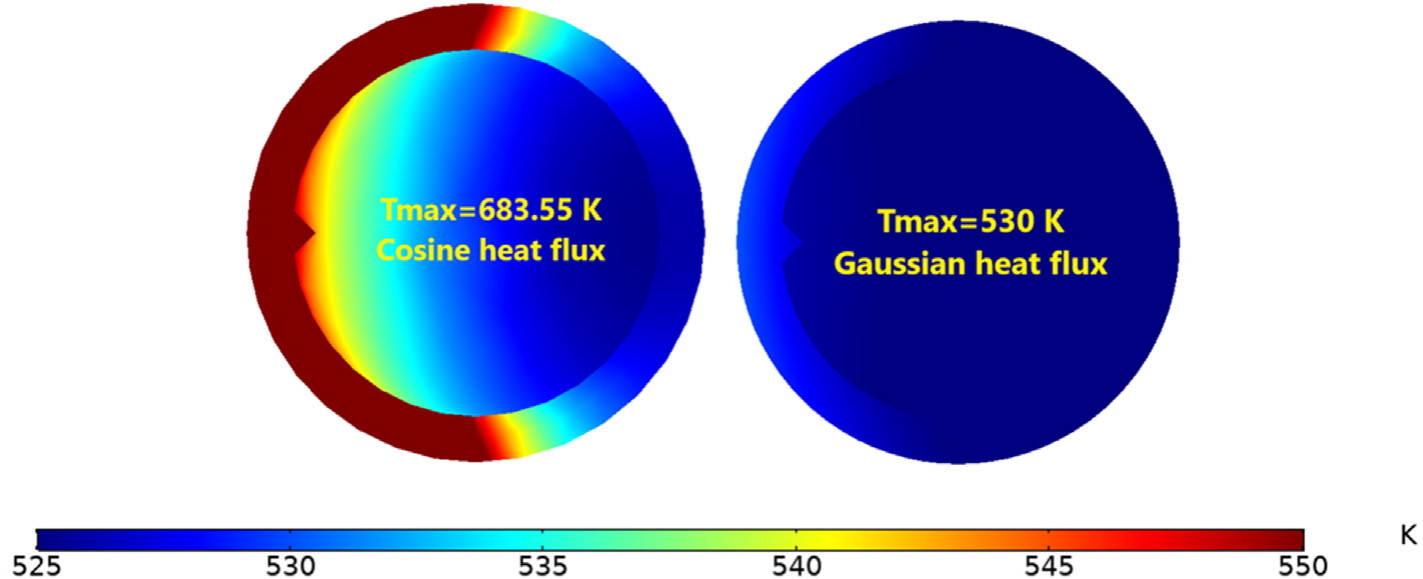
Maximum temperature for Cosine and Gaussian heat fluxes.

**Figure 14:**
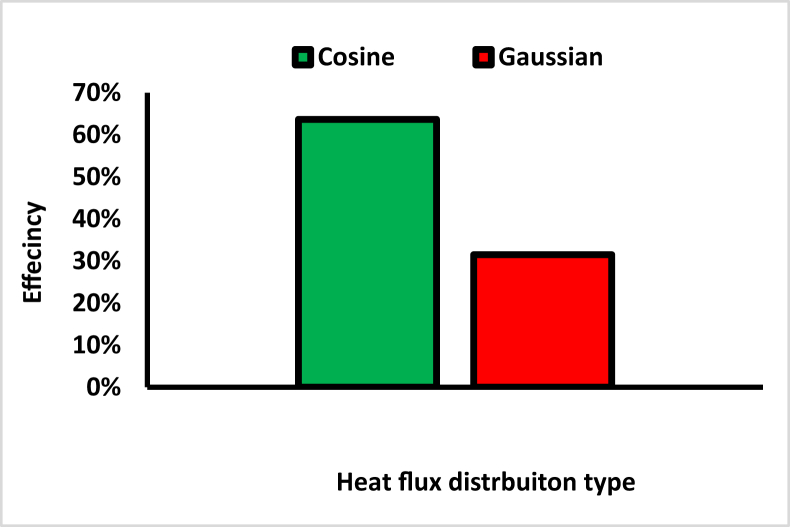
Receiver thermal efficiency with different heat flux distrbuiton.

The maximum temperature on the outer surface of the cosine distribution is higher than the Gaussian distribution by 29%, as shown in Figure 13, due to the higher spillage losses with the Gaussian heat flux distribution. Spillage loss is caused by the energy destined for the receiver not reaching the receiver's area. To evaluate the spillage losses, the receiver thermal efficiency was considered (see Eq. 10), which is reflected as thermal power transferred to the molten salts (Q_con_) to the radiation power that reaches the receiver (Q_Rad rec_) [27]_._ Accordingly, the $\eta_{R}$ was higher for the cosine distribution by 102%, as shown in Figure 14.

(10) $$\eta_{R}=\frac{{\dot{Q}}_{\text{Conv}}}{{\dot{Q}}_{\text{Rad}\;\text{rec}}}$$

**Figure 15:**
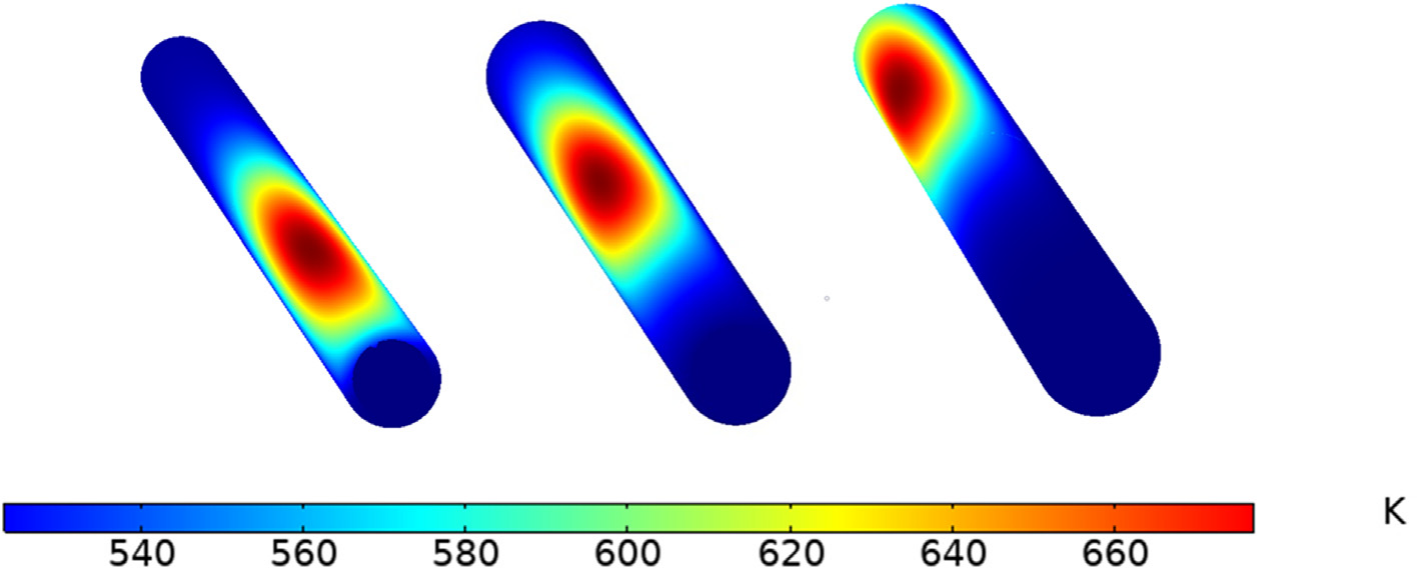
Different angle spans of Gaussian non-uniform heat flux distribution.

**Figure 16:**
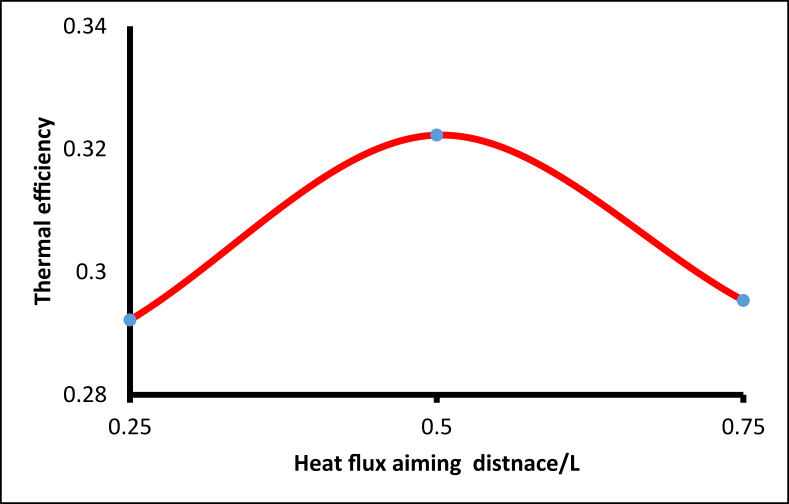
Receiver thermal efficiency with different aiming angles.

To evaluate the overall performance with a different aiming point, when the heat flux was distributed in a Gaussian manner, three aiming points were selected at various angles along the receiver length (x = 0.25L, 0.5L, and 0.75L), as shown in Figure 15. The thermal efficiency (Eq. 8) when aiming at the middle point of the receiver was higher by 10% compared with the lower aiming point at (x = 0.25L), and higher by 9% compared with the upper aiming point (x = 0.75L), as illustrated in Figure 16.

### 3.3 The effect of different tube thicknesses

To study the effect of receiver thickness (t) on the heat transfer performance, first, the internal diameter was fixed and the outer diameter was changed. Different receiver tube thickness values were chosen as t = 2.2 mm, 2.0 mm, 1.2 mm, and 0.25 mm [12]. Decreasing the receiver's thickness by fixing the internal diameter meant decreasing the external diameter (D_out_). Decreasing the receiver's thickness by keeping the external diameter fixed meant increasing the internal diameter, for both cases of decreasing (D_out_) or increasing (D_in_). The wall temperature and heat flux fell and the heat transfer rate would increase [28], this is reflected by the maximum temperature on the outer wall surfaces.

**Figure 17:**
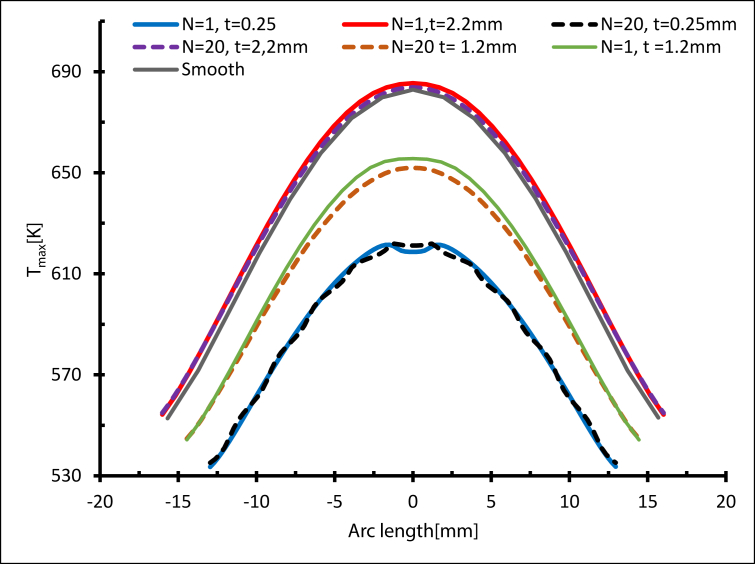
Temperature distribution from the heated side, Re = 14000.

**Figure 18:**
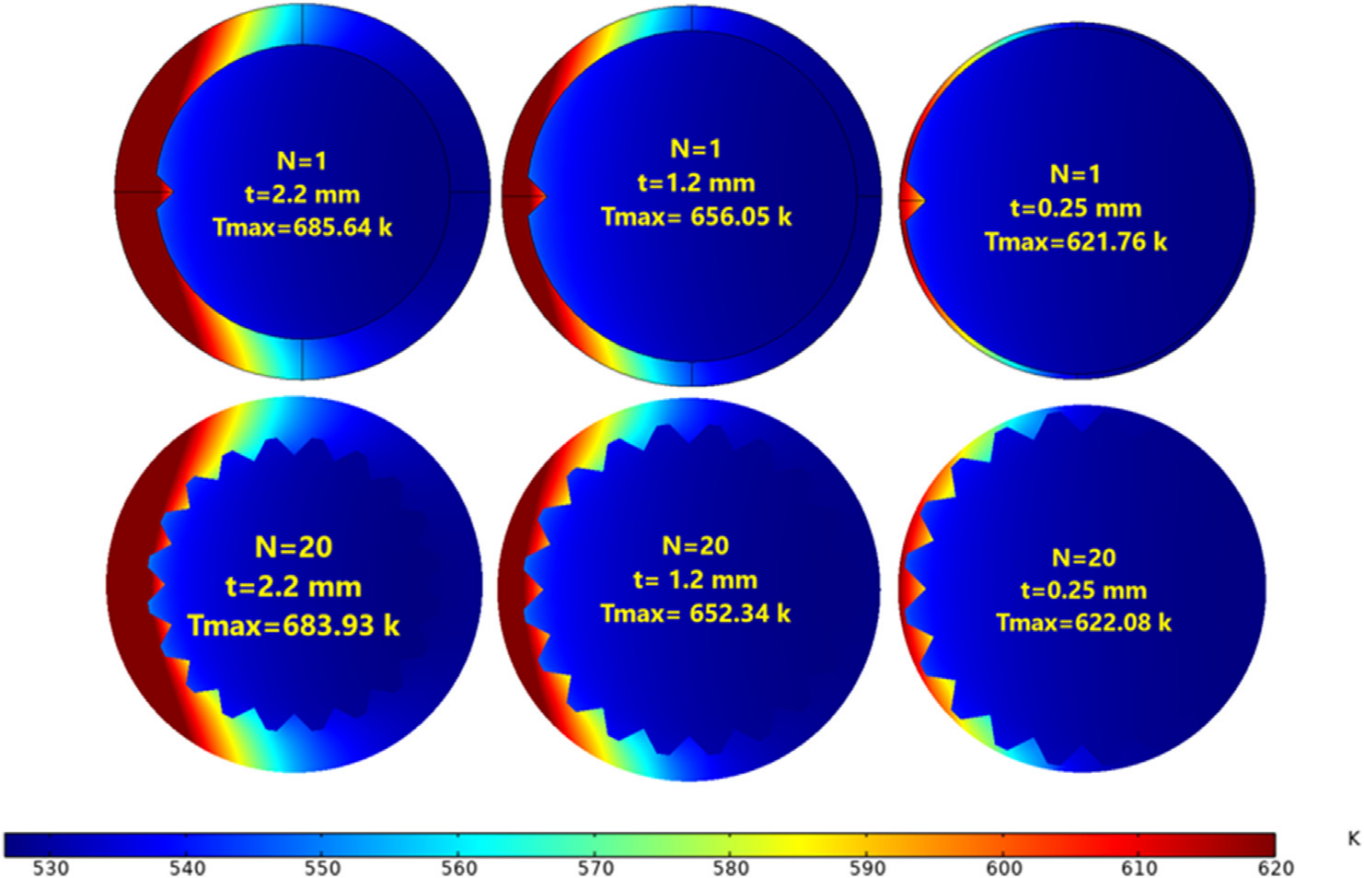
The maximum temperature at Re = 14000.

Figure 17 shows the maximum temperature distribution near the heated exit circumference at Re = 14000 with different tube thicknesses. The maximum temperature (T_max_) meant a lower heat transfer rate, while as the thickness decreased the maximum temperature fell but the heat transfer rate rose. Even though the fins' impact was not significant here, the thickness effect is quite obvious. T_max=_ 685.64 K for N = 1 and t = 2.2 mm was the highest temperature, while the lowest was T_max=_ 621.76 K for N = 1 and t = 0.25 mm. A summary of the maximum temperature is shown in Figure 18.

**Figure 19:**
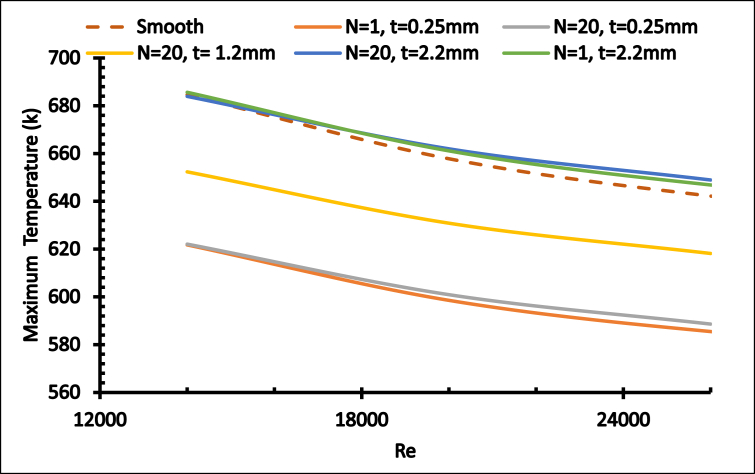
Maximum temperature with different Re. with fixed internal diameter.

Figure 19 explains the relationship between Re and T_max_. Obviously, the Reynolds number is inversely proportional to the maximum temperature. The optimum case was determined with higher Reynolds numbers when N = 1 and t = 0.25mm.

**Figure 20:**
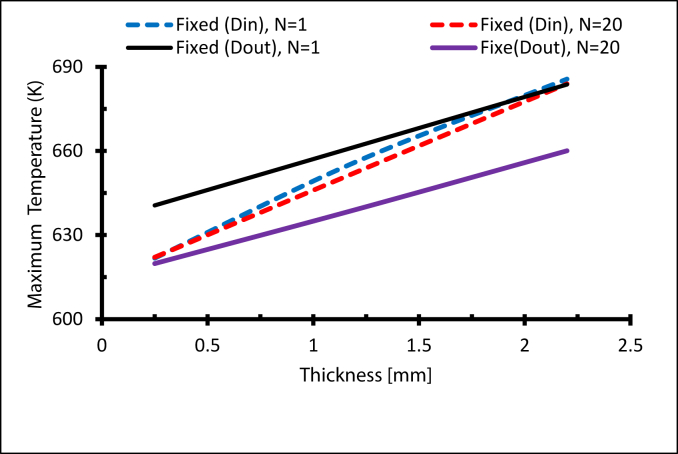
The maximum temperature at Re = 14000 with different receiver thicknesses.

Increasing the receiver diameter raises solar flux levels on the receiver but simultaneously decreases the convection of heat from the internal surface [13]. A small tube thickness provides a lower maximum temperature since the thermal resistance grows with a larger tube thickness [28]. Figure 20 summarises the effect of receiver thickness. The maximum temperature in the receiver rises as the thickness increases [14], while using thin tube walls reduces the temperature gradient and, therefore, reduces thermal stress.

### 3.4 The effect of receiver material

Receiver tubes are exposed to a harsh working environment. Selecting the receiver material required careful consideration to mitigate the extreme operating conditions and to ensure the receiver's durability, reliability and integrity during its lifecycle. The essential requirements are high resistance to corrosion, optimal physical and mechanical properties, great fabricability, and a greater allowance for flux density [29].

A better tube can be found with high thermal conductivity [28]. Nonetheless, a better-performing receiver can only be achieved using a material with mechanical strength and structural stability under high temperatures, such as alloys. Alloy metals reduce thermal conductivity and raise thermal resistance. A lower thermal conductivity implies a reduction in heat losses. Five different materials were tested, as shown in Table 1. The target was to identify the tube material that could survive the high temperatures by keeping the maximum temperature on the outer surfaces and displaying high resistance.

**Table 1:** Physical properties of receiver material [14].

Material name	ρ [Kg/m^3^]	C_p_ [W/m.K]	K [J/kg.K]
Alloy 625	8440	505	16.4
Alloy 800 H	7940	460	18.3
SiC	3210	1200	70
AlSi12	2661	939	181
Copper	8940	450	340

**Figure 21:**
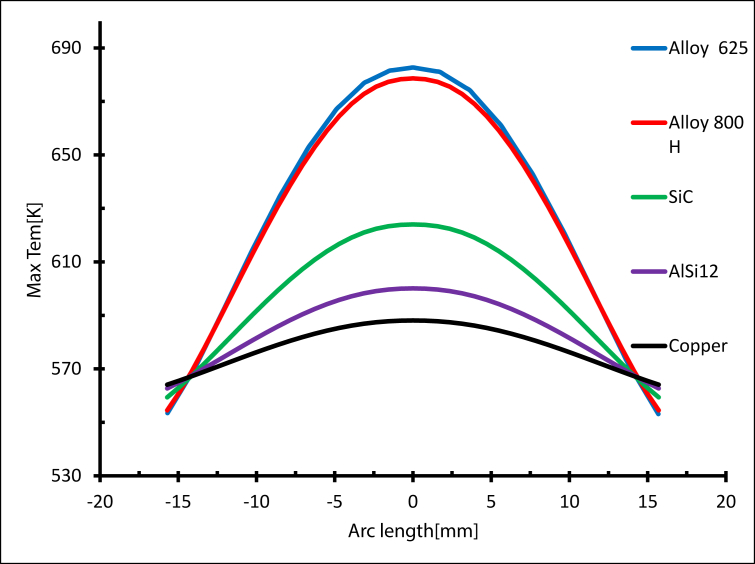
The maximum temperature with different receiver materials at Re = 14000.

**Figure 22:**
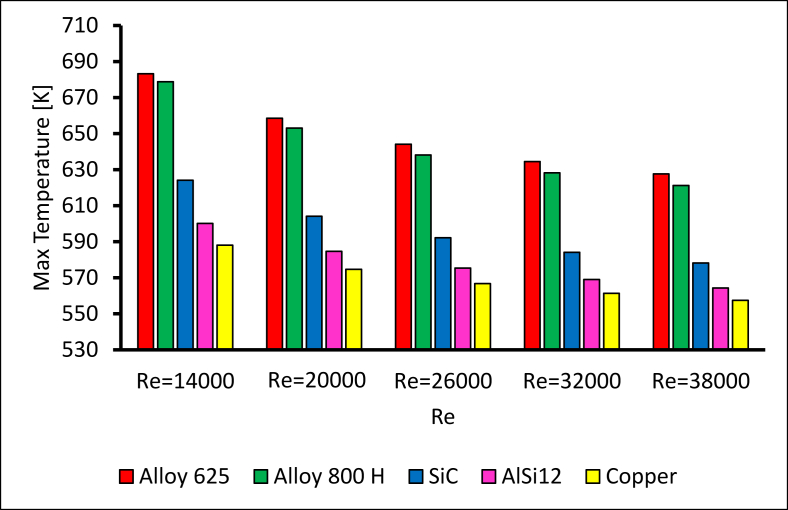
The maximum temperature for different receiver materials.

**Figure 23:**
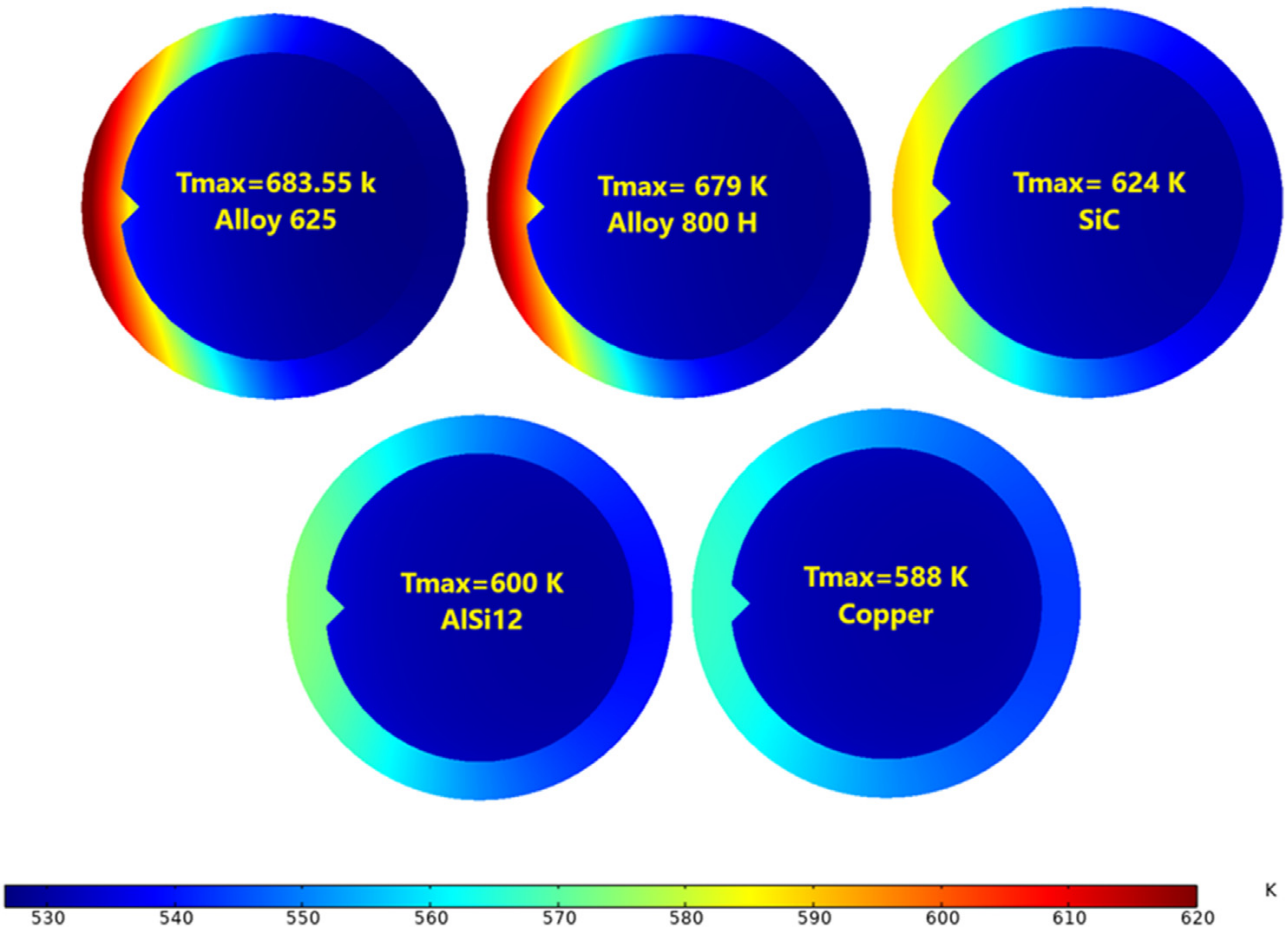
Temperature contour with different receiver materials at Re = 14000.

Figure 21 demonstrates the maximum temperature of the outer surface near the exit with Re = 14000. Alloy 625 was distinguished as the most appropriate choice for solar receiver manufacturing [14] with high thermal resistance. When Re = 14000, the differences in the maximum temperature between alloy 625 and alloy 800H, SiC, AlSi12, and copper sequentially are 1%, 9%, 14%, and 16%. Furthermore, when Re = 38000, the differences are 1%, 8.5%, 11%, and 12.6% respectively. Figure 22 summarises the maximum temperature of the studied receiver's material for different Reynolds numbers. Figure 23 displays the temperature contour at the receiver exit.

### 3.5 The effect of different heat transfer fluids

#### 3.5.1 Nanoparticles with solar salt as a base fluid

For heat transfer fluid selection, the nitrate salt is a mixture of sodium nitrate by 60% weight (NaNO_3_) and potassium nitrate by 40% weight (KNO_3_) was selected as a base fluid. It has a low vapour pressure and stable in air. Properties of fluid are found from Eqs. (11), (12), (13), and (14) [30], where T is the bulk temperature = Tin [K].

Density as a function of temperature:

(11) *ρ (kg/m*^*3*^*)* = *2090* − *0.636* × *T (°C)*

Specific heat as a function of temperature:

(12) *C*_*p*_*(J/kg °C) = 1443 + 0.172 × T (°C)*

Absolute viscosity as a function of temperature:

(13) *μ (mPa.sec) = 22.714−0.120×T (°C) +2.281 × 10^−4^× (T (°C))*^*2*^*− 1.474 × 10*^*−7*^*× (T (°C))*^*3*^

Thermal conductivity as a function of temperature:

(14) *k (W/m °C) = 0.443 + 1.9 × 10*^*−4*^*× T (°C)*

Nanoparticles specifications:

Aluminium oxide Nanopowder (gamma)-Hydrophilic Nanoparticles Al_2_O_3_ Purity: +99%. APS(average particle size): 20nm - 50%, 20nm- 25% < 20nm, 25wt%>20nm, max 2–3% 50nm. Nanoparticles Al_2_O_3_ making method: High-temperature Combustion Method [31]. Concentration (wt.%):0.0,0.016,0.063,0.125,0.25,0.5. Nanoparticle thermal conductivity at room temperature = 36 (W/m K) [32].

Nanofluid physical properties are obtained from the Eqs. (15), (16), (17), and (18) [19, 33, 34]:

(15) $$\rho_{\text{nf}}=\varphi \rho_{\text{np}}+\left(1-\varphi \right)\rho_{\text{b}f}$$

(16) $$\frac{k_{\text{nf}}}{k_{\text{bf}}}=\frac{k_{\text{np}}+2k_{\text{bf}}-2\varphi \left(k_{\text{bf}}-k_{\text{np}}\right)}{k_{\text{np}}+2k_{\text{bf}}+\varphi \left(k_{\text{bf}}-k_{\text{np}}\right)}$$

(17) $$\mu_{\text{nf}}=\mu_{\text{bf}}\frac{1}{{\left(1-\varphi \right)}^{2.5}}$$

(18) $$C_{{\text{p}}_{\text{nf}}}=\left(1-\varphi \right)C_{{\text{p}}_{\text{bf}}}+\varphi C_{{\text{p}}_{\text{np}}}$$

where:

*ρ*: density (kg/m^3^), k: thermal conductivity (W/m K), μ: dynamic viscosity (Pa.sec), C_p_: specific heat (J/kg K) nf: nanofluid, np: nanoparticles, bf: base fluid, f: fluid, φ : the nanoparticle volume concentration.

**Figure 24:**
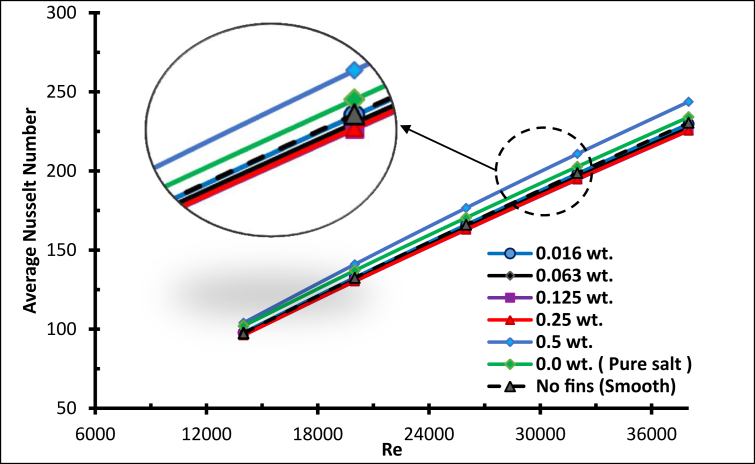
Average Nusselt number for nanofluid with different weight ratios.

Figure 24 below shows the average Nusselt number with Reynolds numbers. It is clear that as the Reynolds number increases, the average Nusselt number does also, which enhances the heat transfer process [19]. In addition, for all concentrations of molten salt-based nanofluids and pure molten salt, the heat transfer performance is better with higher Reynolds numbers [35]. As the concentrations of nanoparticles increase, the heat transfer does not increase gradually [19]. The effect of specific heat was dominant for concentrations (0.016, 0.063, 0.125 and 0.25) since the average Nusselt number for the mentioned concentration was lower than that for the pure salt, and the specific heat was inversely proportional to the concentration. As the concentration decreased, the average Nusselt number rose, for concentrations <0.25 wt.%.

However, for 0.5wt. % and above, the thermal conductivity played a role in enhancing the heat transfer against the specific heat, which is consistent with [36], where the optimal increase in heat transfer was found with 0.7 wt.% of Al_2_O_3_ NPs, taking into account that raising the concentration above 0.5 wt. % harms the specific heat capacity [37], and the enhancement of the specific heat of molten nitrate salts is always concentration-dependent.

**Figure 25:**
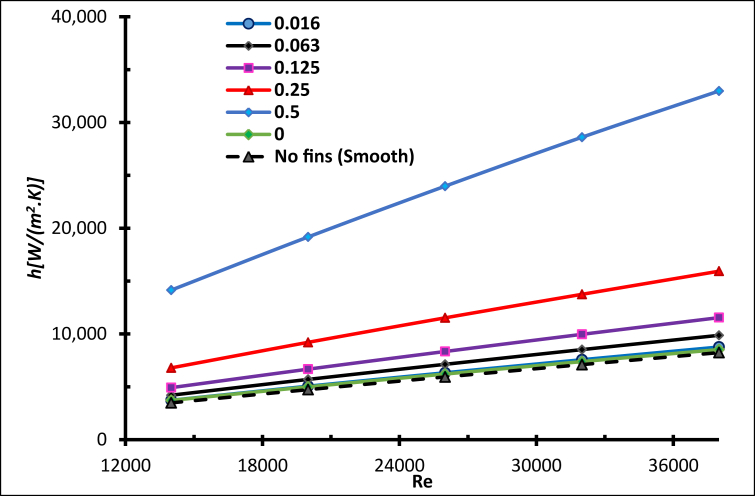
Heat transfer coefficient with different weight ratios.

The mass concentration of AL_2_O_3_ nanoparticles has a remarkable influence on the heat transfer coefficient; the fluid convection after adding nanoparticles to the molten salt has been improved [38]. For all Molten Salt-Based Nanofluids (MSBN) the heat transfer coefficient is better compared with pure molten salt, as shown in Figure 25 When the Reynolds number increased, the coefficient of convective heat transfer raised significantly, since the thickness of the thermal boundary layer was minimised by increasing the turbulence strength [25].

**Figure 26:**
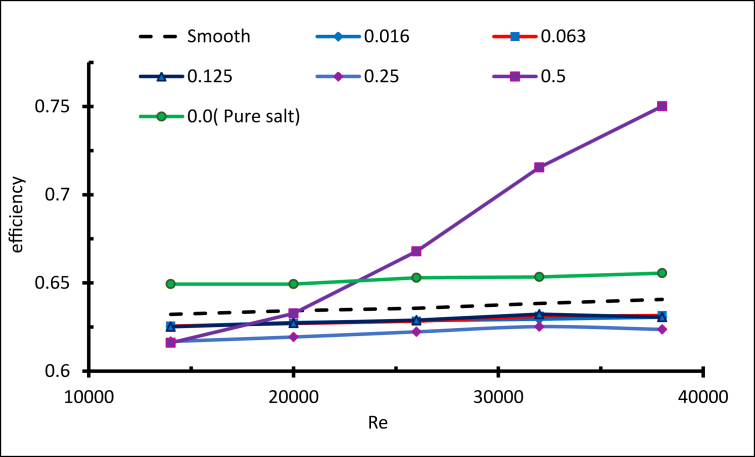
Thermal efficiency with different weight ratios.

The effect was remarkable when concentration was 0.5 wt. %, where nanoparticles increased the thermal conductivity of the mixture and the high energy exchange process was initiated. To evaluate the addition of nanoparticles to molten salt, the thermal efficiency is explained in Figure 26 The specific heat of the nanofluid was generally lower than pure molten salt. This is reflected by the thermal efficiency being lower than the pure salt for all concentrations below 0.5 wt.%. However, for the concentration 0.5 wt.%, the thermal efficiency for Re < 23000 was less than the pure salt, but as the Reynolds number increased the efficiency increased, to reach a maximum when Re = 38000, which is 14% more than pure molten salt.

**Figure 27:**
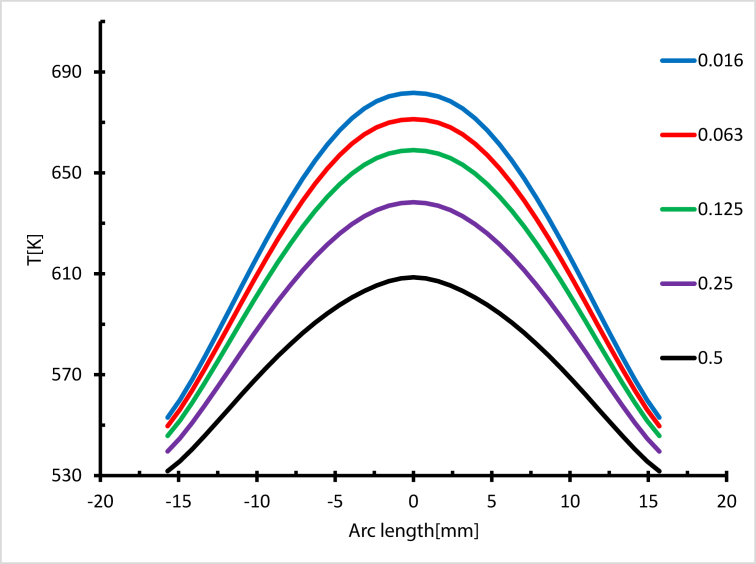
Maximum Temperature distribution from the heated side, Re = 14000.

**Figure 28:**
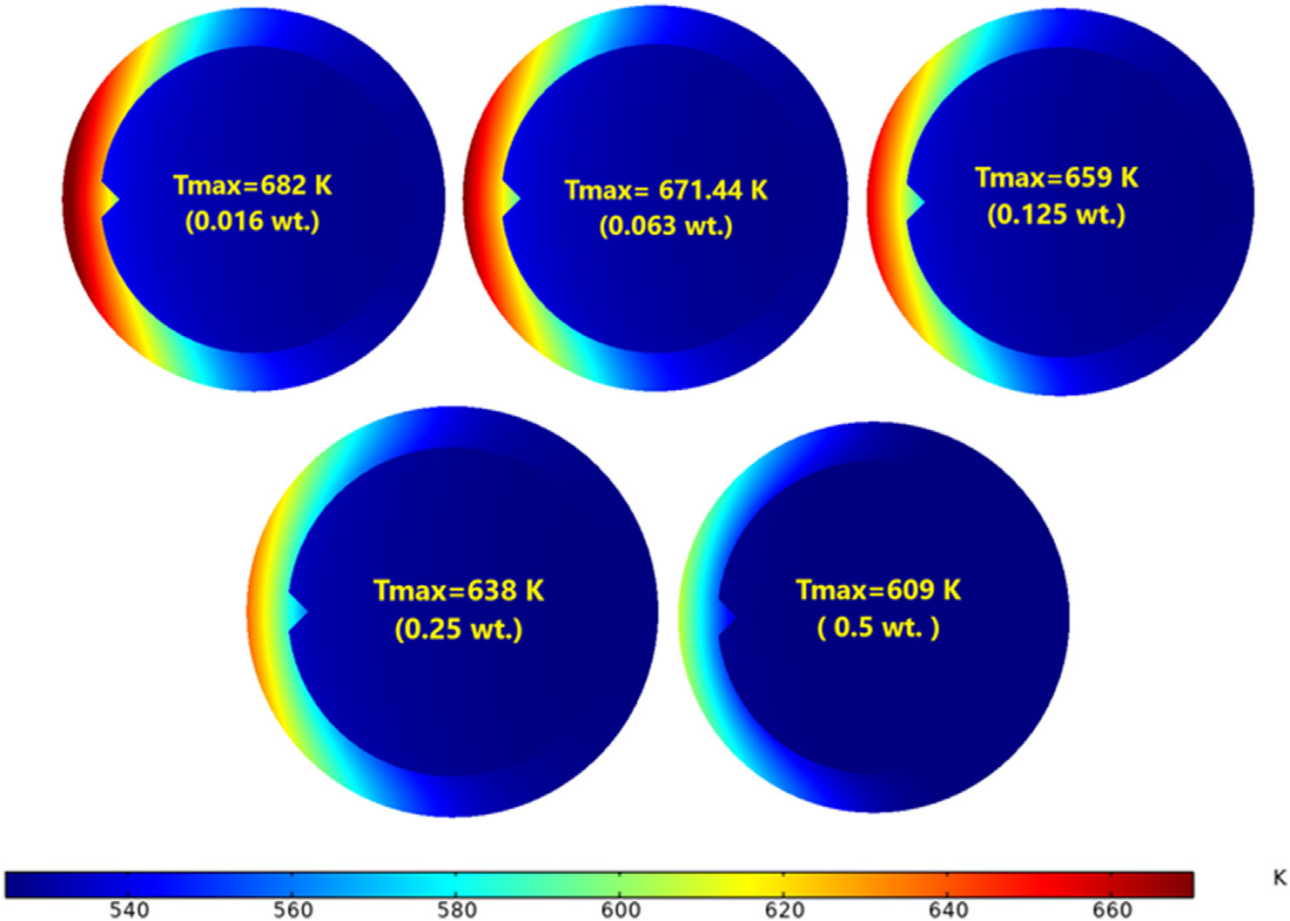
Temperature contour at Re = 14000.

Figure 27 shows the maximum temperature near the exit on the heated side at Re = 14000. The heat transfer process was enhanced by adding nanoparticles, and as the concentration increased up to 0.5 wt.%, the maximum temperature decreased. This was due to the receiver's outer surface being cooled by heat transfer fluid, which has a high thermal conductivity. The maximum temperature decreased by 12 % with a concentration of 0.5 wt.%, but the reduction in the maximum temperature was 0.2%, 1.8%, 3.7% and 7.1% for 0.016, 0.063, 0.125 and 0.25 wt.% respectively, as shown in Figure 28, which represents the temperature contour at the exit and highlights the maximum temperature of the receiver.

**Figure 29:**
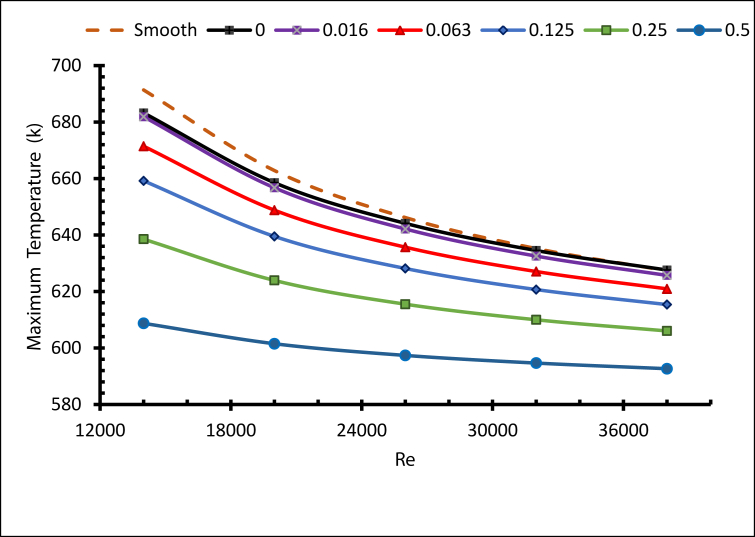
Maximum temperature with different weights ratios.

In summary, the heat transfer process with nanofluid was enhanced with a concentration near 0.5 wt. %, and the maximum temperature reduced with the increases in concentration. As the Reynolds number fell, the cooling of the outer surface of the receiver rose, while and the maximum temperature decreased, as shown in Figure 29.

#### 3.5.2 The effects of liquid sodium and Hitec as heat transfer fluids

Restricting temperature range of molten salt inspired many scientists to discover different working fluids, such as liquid sodium. Liquid sodium can reach high working temperatures as a heat transfer fluid to provide a more robust system. On the other hand, a rise in working temperature means an increase in thermal stresses. The design of a solar receiver with the addition of internal triangular fins and which uses liquid sodium has been considered a promising solution for high operational temperatures.

For liquid sodium Eqs. (19), (20), (21), and (22) are used to evaluate the physical properties, for Hitec (7% NaNO_3_, 53% KNO_3_, 40% NaNO_2_) it is obtained from Eqs. (23), (24), (25), and (26) [15, 39, 40, 41].

(19) $$\rho =219+275.32\left(1-T/2503.7\right)+{511.58\left(1-T/2503.7\right)}^{0.5}$$

(20) $$k=124.67-0.11381\cdot T+5.5226\times 10^{-5}\cdot T^{2}-1.1842\times 10^{-8}\cdot T^{3}$$

(21) $$c_{p}=1658.2-0.84790\cdot T+4.4541\times 10^{-4}\cdot T^{2}-2.9926\times 10^{6}\cdot T^{-2}$$

(22) ln(μ)=−6.4406−0.3958ln(T)+556.835/T

(23) ρ=−0.74(T−273.15)+2084

(24) $$k=0.411+4.36\times 10^{-4}\left(T-273.15\right)+1.54\times 10^{-6}{\left(T-273.15\right)}^{2}$$

(25) $$c_{p}=1560-\left(T-273.15\right)$$

(26) $$\mu =10^{2.7374}{\left(T-273.15\right)}^{-2.104}$$

Molten salt is stable as a heat transfer fluid up to temperatures of 595 °C, and the temperature at which the decomposition of molten salt starts is between 600 to 700 °C. It is challenging to operate a solar receiver with molten salt with a peak heat flux more than 0.8 MW/m^2^. However, because of the high thermal conductivity of liquid sodium, the peak heat flux operates up to 2.5 MW/m^2^ [42]

**Figure 30:**
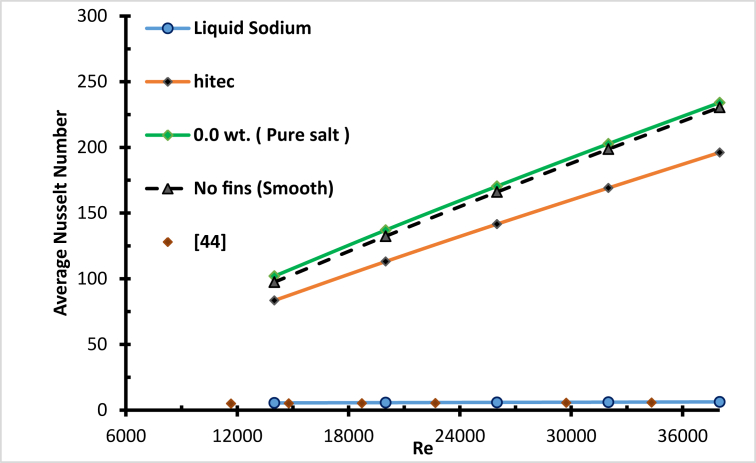
Validating liquid sodium and Nusselt number, compared with [44].

**Figure 31:**
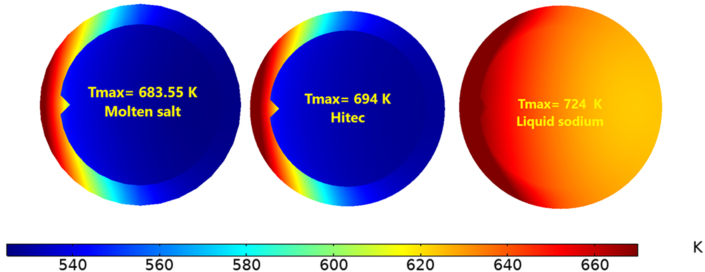
Maximum temperature with different heat transfer fluid, at Re = 14000.

Even though the Nusselt number was lower for sodium liquid by about 1760 % compared with molten salt and 1420 % lower compared with Hitec (as shown in Figure 30), this is due to the higher thermal conductivity and the lower wall temperature of liquid sodium. However, the radiation heat loss was lower due to a low wall temperature, and the thermal efficiency is higher [43]. As a result, the maximum operating temperatures were 724 K, 694 K, 684 K for liquid sodium, Hitec, and molten salt, respectively, as shown in Figure 31.

**Figure 32:**
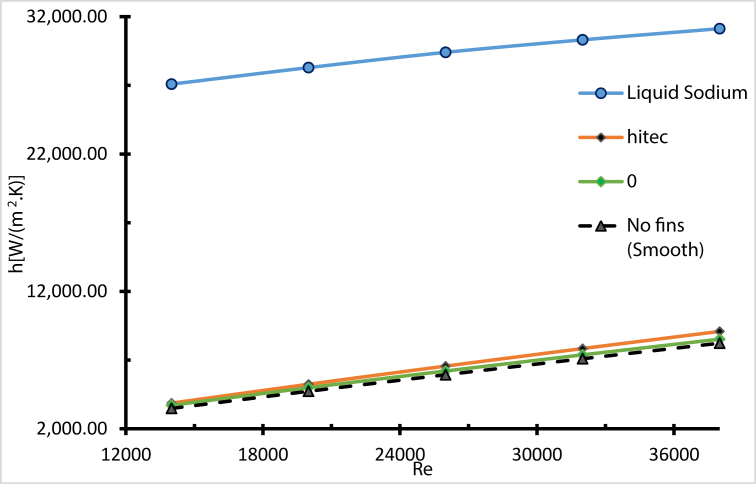
Heat transfer coefficient with different heat transfer fluids. Effect of different HTF with same flow rate.

To further understand the effect of using Liquid sodium, heat transfer coefficient has been plotted in Figure 32 The heat transfer coefficient of liquid sodium is greater than the other fluids, which is explained by the highest thermal conductivity of sodium. The results show that even turbulence is necessary, but thermal conductivity still dominates [15].

**Figure 33:**
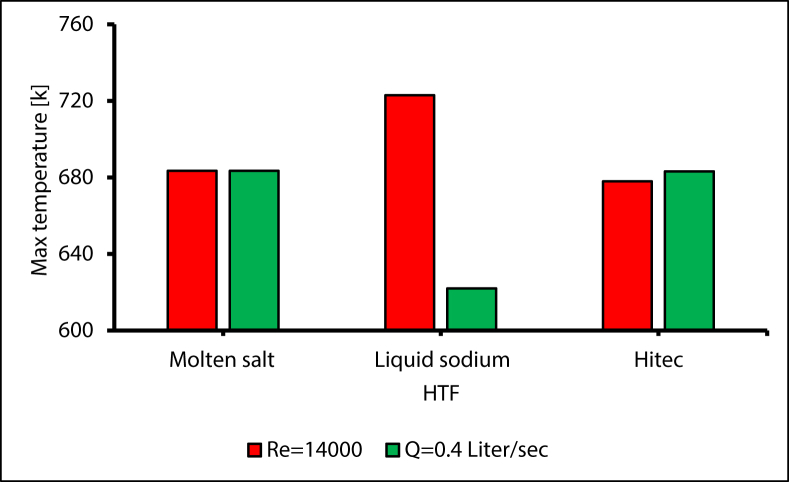
Maximum temperature with different HTF, with constant Re & Q.

To study different HTF effects with the same flow rate, the maximum temperature was plotted for a flowrate equal to 0.4 L/sec, as shown in Figure 33 The maximum temperature fell by 10% for the liquid sodium compared with molten salt, and by 0.05% for Hitec compared with molten salt. Similarly, the Prandtl number was far smaller for liquid sodium compared with molten salt, which indicates that thermal diffusivity is much higher than momentum diffusivity. As a result, the thickness of the thermal boundary layer is less than the velocity layer, which makes the convective heat transfer coefficient substantially higher, and the maximum temperature lower. Moreover, the effect of thermal inertia is smaller for liquid sodium on the heat transfer process since it has less (*ρ·Cp*) [45].

**Figure 34:**
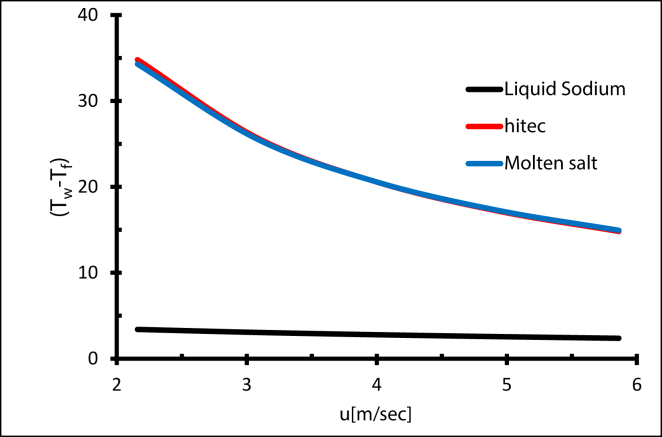
Temperature gradient for different HTF.

Using liquid sodium helps to reduce the heat losses from the receiver by reducing the temperature gradient between the receiver wall temperature and the mean fluid temperature [46], (as illustrated in Figure 34). This reduced the thermal stress in the receiver tube, and then decreased the deflection of the receiver. Generally, increasing the overall efficiency is achievable using liquid metal as the heat transfer fluid.

### 3.6 Thermal stress

The solar receiver tube experiences thermal stress due to the non-homogeneous change in temperature on the outer surfaces. It is crucial to consider the thermal expansion occurring when a solar receiver is heated. The maximum heat flux on the outer side of the receiver tubes is restricted to extend the life of the receiver. The spread of heat flux induced by inhomogeneous radiation eventually impacts the distribution of temperature on the receiver tube. Thermal stresses and temperature gradients are the outputs for the periodic and non-uniform heat flux distribution [24].

**Figure 35:**
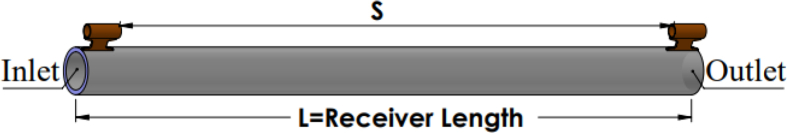
Receiver length with distance between clips.

Clips were welded to the receiver's tubes and used to direct the receiver along its axis as the tubes expanded and shrank throughout the heating process. Contact with cold clips may allow a heat leak or produce cold spots in the morning that will freeze the salt and stop the system [47]. In this study, the effect of adding internal fins was investigated with consideration of the distance between clips (S), as shown in Figure 35, and by obtaining the thermal stresses and the displacement of the tube.

The same receiver length (L) was selected as the Gemasolar receiver, and was 10 m long, and the distance (S) between clips of 2 m [24]; the clips were simulated as fixed points of the tube.

**Figure 36:**
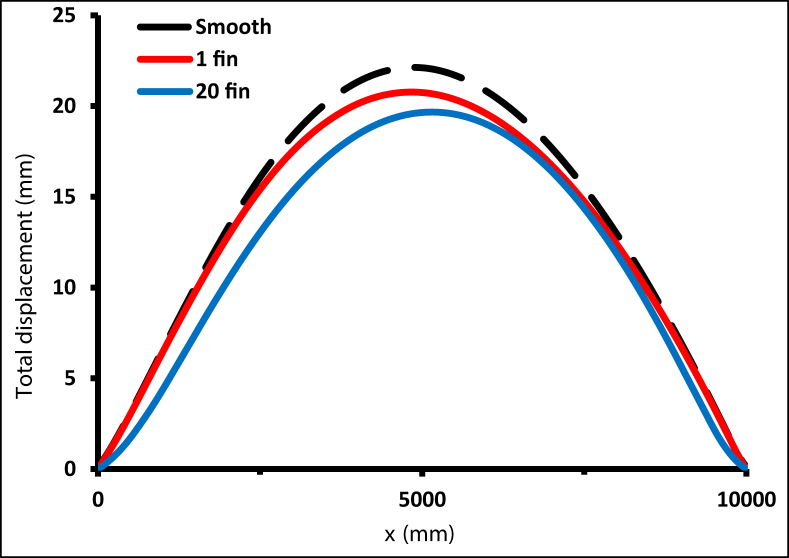
Total displacement of the receiver with ends encastred.

The leading cause of thermal stress is the wide circumference temperature gradient. When a small temperature difference in the radial direction occurs, high thermal stress can occur [48]. The first set of conditions assumed that the two ends of the receiver tube were encastred and no clips were used. As shown in Figure 36, the total displacement (in y and z) was higher for the smooth tube compared with a finned tube. Adding fins reduced the deformation of the receiver, while adding one fin reduced the total displacement by 6.5%, and adding 20 fins reduced the displacement by 12.5%.

**Figure 37:**
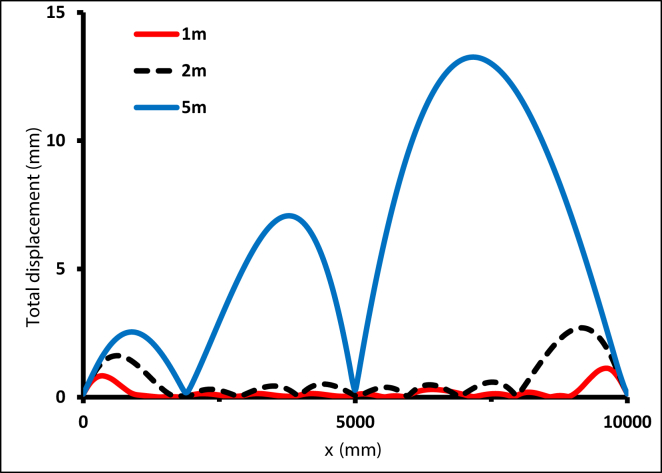
Total displacement with distance between clips = 1, 2, and 5m.

For the second set of conditions, the distance between clips was selected as 1 m, 2 m, and 5 m, as illustrated in Figure 37 The inclusion of clips decreased bending significantly, and as the distance between clips decreased, the displacement of the tube decreased. The maximum displacement when S = 1 m was 1.125 mm, and it increased up to 2.7 mm when the S = 2 m. It then reached the highest value, 13.25 mm, when S = 5 m.

## 4 Conclusion

The optimisation of a solar receiver tube was studied numerically from different aspects after strengthening the tube by adding internal fins. Adding one triangular fin longitudinally has a significant effect, as it increases the Nusselt number by 4% and thermal efficiency by 2.5%, compared with a smooth tube. Receiver efficacy is affected by heat flux distribution, and it can be higher by 102% for the cosine heat flux distribution compared to the Gaussian distribution. Changing the heat flux aiming point on the receiver from the first quarter to the middle point increases the receiver efficiency by 10%. As the thickness of the receiver tube decreases, the heat transfer rate rises. A thickness of 0.25 mm provides the best heat transfer performance. Alloy 625, as a receiver material which can keep the maximum temperature on the surface, can be considered appropriate as a solar receiver material. The heat transfer process improved with nanofluid which has a concentration of around 0.5 wt. An improvement in the overall efficiency is achieved using liquid sodium as the heat transfer fluid, which has a 1000% higher heat transfer coefficient. Adding fins reduces the deformation of the tube, as well as reducing the thermal stresses. Adding one fin reduces the displacement by 6.5% compared with the smooth tube. As the distance between clips decreases, the displacement decreases. Further investigation is recommended to determine the peak heat flux using internal fins.

## Declarations

### Author contribution statement

Hashem Shatnawi: Conceived and designed the experiments; Performed the experiments; Analyzed and interpreted the data; Contributed reagents, materials, analysis tools or data; Wrote the paper.

Chin Wai Lim, Firas Basim Ismail & Abdulrahman Aldossary: Conceived and designed the experiments; Contributed reagents, materials, analysis tools or data.

### Funding statement

This research did not receive any specific grant from funding agencies in the public, commercial, or not-for-profit sectors.

### Data availability statement

Data will be made available on request.

### Declaration of interests statement

The authors declare no conflict of interest.

### Additional information

No additional information is available for this paper.
